# In situ neutron imaging of lithium-ion batteries during heating to thermal runaway

**DOI:** 10.1038/s41598-023-49399-1

**Published:** 2023-12-12

**Authors:** Hiroshi Nozaki, Hiroki Kondo, Takenao Shinohara, Daigo Setoyama, Yoshihiro Matsumoto, Tsuyoshi Sasaki, Kazuhisa Isegawa, Hirotoshi Hayashida

**Affiliations:** 1 Toyota Central Research and Development Laboratories Inc., 41-1 Yokomichi, Nagakute, Aichi 480-1192 Japan; 2 Japan Atomic Energy Agency (JAEA), Tokai, Ibaraki 319-1195 Japan; 3 Comprehensive Research Organization for Science and Society (CROSS), Tokai, Ibaraki 319-1106 Japan

**Keywords:** Imaging techniques, Batteries, Imaging techniques

## Abstract

Lithium-ion batteries (LIBs) have become essential components that power most current technologies, such as smartphones and electric vehicles, thus making various safety evaluations necessary to ensure their safe use. Among these evaluations, heating tests remain the most prominent source of safety issues. However, information on the phenomena occurring inside batteries during heating has remained inaccessible. In this study, we demonstrate the first in situ neutron imaging method to observe the internal structural deformation of LIBs during heating. We developed an airtight aluminium chamber specially designed to prevent radioactive contamination during in situ neutron imaging. We successfully observed the liquid electrolyte fluctuation inside a battery sample and the deformation of the protective plastic film upon heating up to thermal runaway. Hence, this work provides the foundation for future investigations of the internal changes induced in batteries during heating tests and experiments.

## Introduction

With battery-based electric vehicles (EVs) at the forefront of almost every major car manufacturing industry worldwide, the increasingly rapid technological evolution of batteries during the last decade has enabled the widespread adoption of EVs in modern society. To date, lithium-ion batteries (LIBs) have mainly been utilised as energy-storage devices in EVs owing to their high power and energy density. Despite their tremendously advantageous use in powering vehicles, LIBs require significant safety measures upon their disposal for recycling or repurposing within the framework of carbon neutralisation^1–5^. Several tests are used to evaluate the safety of LIBs, including nail penetration, overcharge, overdischarge, short circuit, and thermal abuse tests^6–8^. The nail penetration test assesses a battery’s internal short circuit mechanism, which is affected by contamination during the manufacturing process or by structural defects. The overcharge, overdischarge, and short-circuit tests assess operational complications, such as failures of the control program or wiring errors. The thermal abuse test involves increasing the temperature of the LIB at a constant rate to assess its thermal stability—a fundamental safety factor in used batteries. Thermal stability is evaluated based on whether the LIB enters runaway^9^. Evaluation of the thermal stability of the LIBs involves simultaneous measurements of the temperature and output voltage and observation of battery appearance. The thermal runaway of a battery is caused by the reaction between the charged electrode and the electrolyte solution^10–12^. However, the packaging of battery components in sealed metallic cans or aluminium laminate films has rendered the observation of the inner behaviour of batteries during heating nearly impossible. Therefore, evaluating the actual phenomena that affect thermal stability is based on the evaluation of the thermal behaviour as well as the stability of the individual materials and components of the battery.

Recently, several studies have reported on high-speed observation techniques using synchrotron X-ray radiation to monitor the evolution of internal structural changes and thermal behaviour during a thermal abuse test^13–15^, overcharge test^16^, and nail penetration test^17^. In these studies, the collapse of the electrode was clearly observed just before thermal runaway. X-CT and electrochemical acoustic time-of-flight spectroscopy were performed to analyse the electrode’s delamination^18^. Moreover, a combined analysis using X-CT and accelerating rate calorimetry provided insights into the behaviour during thermal runaway^15^. Although these X-ray-based techniques can reveal valuable information regarding changes in electrodes composed of relatively heavy elements, they remain unsuitable for analysing lithium and electrolytes composed of light elements owing to the low sensitivity of the technique. Notably, the high sensitivity of neutrons to light elements enables the use of a neutron beam as a suitable complementary probe to X-rays. Several studies have reported using neutron beams to investigate internal structural changes during charging and discharging^19–23^. Neutron radiography revealed that excessive electrolyte consumed during the first charge/discharge cycle was attributed to the formation of a solid electrolyte interphase^19^. Gas evolution on the graphite anode was investigated using neutron radiography while charging a battery cell comprising a LiMn_2_O_4_ cathode and a graphite anode^20^. Similarly, the gas evolution in the pouch cells comprising a LiNi_0*.*5_Mn_1*.*5_O_4_ cathode and graphite anode was examined, which revealed that dissolved metal in the electrolyte and decomposition products generates gas during the first cycle^21^. The current rate dependence of the cell expansion behaviour was analysed by observing the cross-section of the battery using neutron radiography. The results indicate that high-rate cycling was the origin of the large cell expansion^22^. Furthermore, the lithium distribution in an 18,650-type cylindrical battery consisting of a LiNi_1/3_Co_1/3_Mn_1/3_O_2_ cathode and graphite anode is dependent on the depth of discharge (DOD), as concluded via neutron radiography^23^. The researchers showed that the lithium distribution decreased in the overdischarged state (DOD = 150%), indicating internal structural degradation.

Neutron experiments provide useful information for assessing the safety of batteries by observing their internal structural changes, particularly in materials comprising light elements. Despite their importance, neutron experiments during heating tests up to thermal runaway have not been conducted thus far owing to technical and radiation safety aspects. From a technical perspective, the experiment requires an intense neutron source because the heating process that leads to thermal runaway of a battery is a dynamical process. In addition, high spatial resolution is necessary to visualise the detailed structure inside the battery. Considering battery reactions, thermal runaway causes radiated gases and particles to erupt; therefore, thermal runaway must be completely confined for radiation safety. In this study, we established a method for the internal visualisation of LIBs during heating tests by developing an in situ neutron imaging observation system.

## Methods

### Lithium-ion battery sample

**Figure 1:**
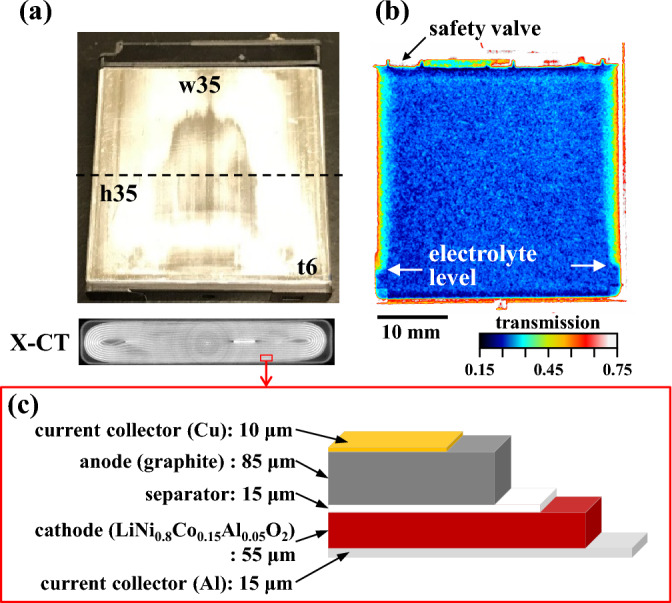
Battery sample and electrode sheet. (**a**) Photographs of the battery sample (top) and X-ray computed tomography image (bottom) of the cross-section indicated with the dashed line. (**b**) Neutron transmission image at room temperature. A safety valve is present on the top left of the battery. (**c**) Schematic diagram of the cross-section structure of the electrode sheet. The values in the figure indicate the thickness of each layer.

Generally, LIBs are commercially available in cylindrical or cuboidal cell shapes. Here, we selected a cuboid-shaped (35 × 35 × 6.1 mm) battery (1 Ah, NP-50, Fuji film) as a sample because the detection of the structural change in neutron radiography is easy for its flatness. The battery comprised a Li(Ni_0*.*81_Co_0*.*15_Al_0*.*04_)O_2_ cathode, a graphite anode, and a separator made of stacked polyethene (PE) and polypropylene (PP) films, confirmed via X-ray diffraction (XRD) and differential scanning calorimetry (DSC). The cathode and anode were coated with Al and Cu foil, respectively. The laminated cathode, separator, and anode were sealed together with the liquid electrolyte into a 0.32-mm-thick Al package. The 6-mm-thick battery is shown in Fig. 1. A fully charged, pristine battery was used as the imaging sample. The charging was performed using constant current and constant voltage (CC–CV) at a charging rate of 0.1 C. The charging duration was 10 h because *x*C corresponds to a charging time of 1*/x* h, where x is the coefficient of charging rate C. The protective plastic film covering the battery was removed to increase neutron transmission. The lead wires were then welded to the positive and negative electrodes to monitor the battery voltage during neutron imaging.

### Neutron imaging

Neutron transmission images were recorded using BL22 (RADEN) in the Materials and Life Science Experimental Facility (MLF) of the Japan Proton Accelerator Research Complex (J-PARC) in Tokai, Japan^24^. The neutron beam power of the J-PARC MLF was maintained at 800 kW throughout the experiments. All images were recorded using a CMOS camera with a resolution of 2048 × 2048 pixels (ORCA-Flash4.0 v3, HAMAMATSU PHOTONICS K.K., Hamamatsu, Japan). The transmitted neutron beam irradiated the ^6^LiF/ZnS scintillator of 100 µm thickness to convert the neutron beam to visible green light. The emitted green light was amplified with an optical image intensifier (C14245-12112-A1, HAMAMATSU PHOTONICS K.K. Hamamatsu, Japan) and detected using the CMOS camera. The details of the camera system are described in prior studies^24,25^. The *L/D* value, which affects the spatial resolution and exposure time, was set to 298, where *L* is the distance between the aperture and scintillator and *D* is the aperture size. The field of view (FOV) was set to 47.6 × 47.6 mm (23.2 µm/pixel). The exposure time was set to 0.48 s, corresponding to 12 neutron pulses, owing to the 25 Hz repetition rate of J-PARC.

The outer chamber was set at the middle stage of the RADEN. The data logger that recorded the battery voltage and temperature as well as the webcam was checked for proper operation before starting the heating experiment. The sample was heated at a rate of 5 °C/min; video recording was started simultaneously with the acquisition of neutron imaging, with data recorded during heating until the thermal runaway was completed.

**Figure 2:**
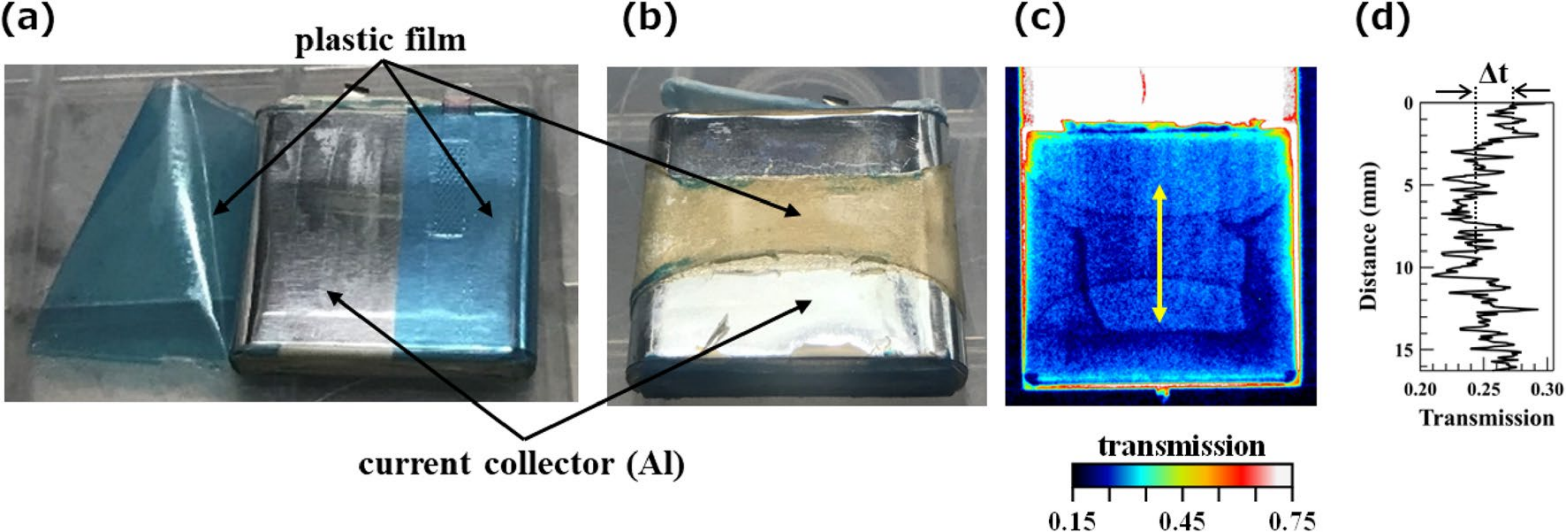
Battery sample before and after heating. (**a**) Photograph of the battery sample with the outer packaging Al foil removed. A blue polypropylene (PP) film is used as an insulator between the outer Al packing foil and the current collector. (**b**) Photograph of the battery sample quenched after heating up to 170 °C. (**c**) Neutron transmission image at 170 °C and (**d**) the line profile along the vertical yellow line in the transmission image.

Additionally, the internal structural changes in the neutron image at 170 °C were evaluated. Figure 2a shows the initial battery sample with the battery can removed. A blue plastic film made of PP on the outer electrode body insulated the body from the battery can. We prepared the battery sample by heating the battery at a rate of 5 °C/min until it reached a temperature of 170 °C. The battery was then cooled rapidly in a furnace. This heat treatment was performed at SOC 0% to ensure experimental safety.

### Data analysis

Imaging data were analysed using ImageJ software^26^. The raw data were converted to transmission maps (*T*) over the entire imaging FOV using

1 $$T\left(t\right)=\frac{{I}_{s}\left(t\right)-{I}_{d}}{{I}_{0}-{I}_{d}},$$

where *t* represents the time from the start of the experiment, and *I* is the two-dimensional (2D) intensity data detected by the CMOS camera. *I*_s_(*t*), *I*_*d*_, and *I*_0_ are the 2D data recorded with the sample, without the neutron beam, and without the sample, respectively. We stacked 10 images to obtain data with an exposure time of 4.8 s for a sufficient signal-to-noise ratio (SNR), although data with shorter exposure times are preferable for analysis to capture the fast changes in the battery. The images were then binned to 1024 × 1024 pixels to obtain accurate statistics.

## Results and discussion

### Airtight heating chamber system

**Figure 3:**
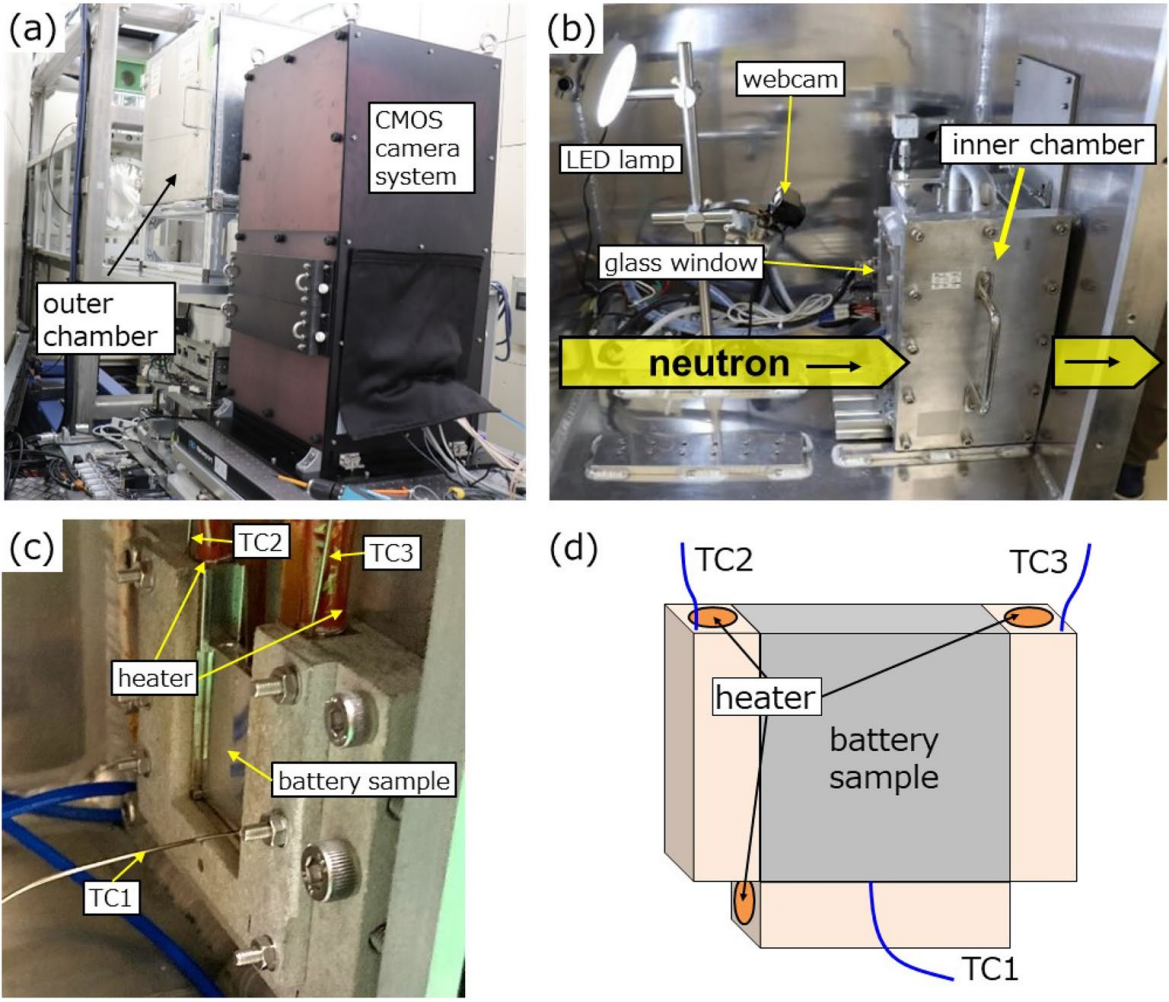
Instrumentation of the heating experiment at BL22 (RADEN). (**a**) Outer chamber and CMOS camera system viewed from downstream, (**b**) inside view of the outer chamber with the webcam and LED lamp, and (**c**) battery sample fixed to the holder inside the inner chamber. (**d**) Three cartridge heaters are placed at both sides and at the bottom of the sample, with three thermocouples (TC) attached to the bottom and top of the heater holder. The TC1 thermocouple measures the sample temperature, whereas TC2 and TC3 are used for controlling the temperature and preventing its excess, respectively.

We developed a novel heating setup specifically designed to prevent the leakage of radioactive materials during neutron experiments and ensure radiation safety. The proposed battery heating system consisted of a dual (inner and outer) aluminium chamber configuration with thicknesses of 12 and 5 mm, respectively (Fig. 3). The total thickness of aluminium (34 mm) in the neutron beam path decreased the neutron intensity by approximately 30%, a considerable loss but acceptable for this study. The inner chamber was designed to be airtight because the internal pressure increased to approximately 140 kPa owing to pyrolysis and vaporisation of the electrolyte after thermal runaway. The chamber was equipped with three cartridge heaters, four thermocouples, a pressure gauge, a glass window, and a leak valve. The heating system was designed to avoid blocking the neutron beam transmitted through the sample, with heaters placed on both sides and at the bottom of the battery sample. The heating rate was set to 5 °C/min using on/off controls, while the temperature fluctuation was suppressed within ± 1 °C. The temperature of each sample was measured using a thermocouple (TC1) placed at the bottom of the sample. Two additional thermocouples (TC2 and TC3) were used to control the heater output and prevent overheating, respectively (Fig. 3). The battery sample was fixed to the inner chamber using the attached thermocouples. A webcam and LED lamp were placed in the outer chamber to monitor the inside of the inner chamber during the experiment.

### Radiography at room temperature

**Figure 4:**
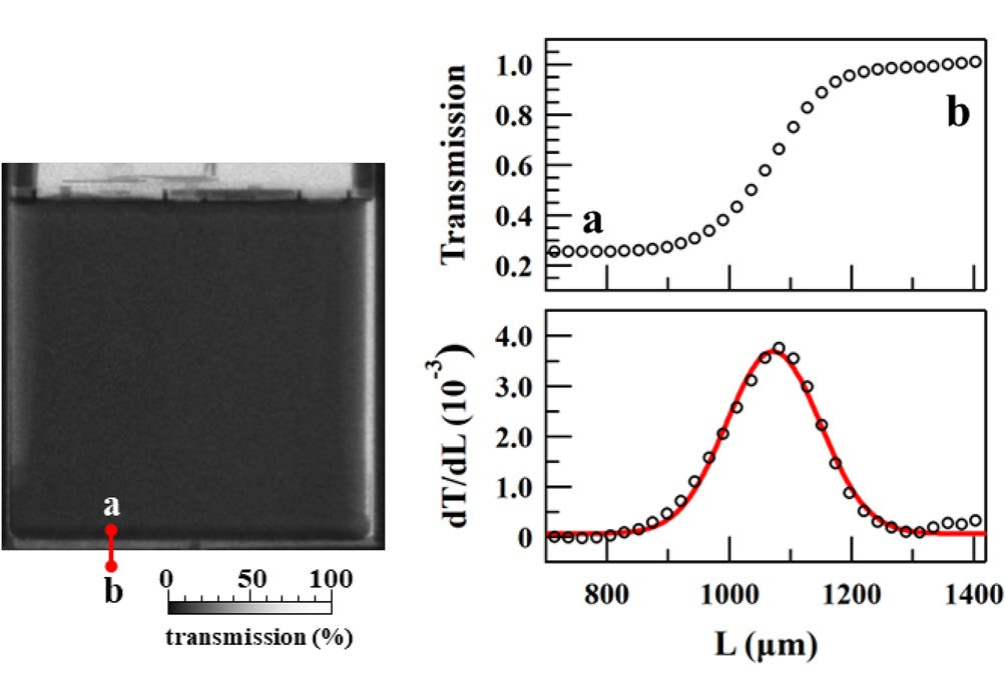
Resolution of the transmission mapping. The spatial resolution was evaluated as twice the standard deviation of a Gaussian function used to fit the differential curve (dT/dL) of the transmission intensity across the bottom edge of the battery. The resolution was estimated at approximately 248 µm.

Figure 1 displays a neutron transmission image of the battery sample, the X-CT image, and the sheet structure of the battery sample. The X-CT image was obtained with a spatial resolution of 140 μm at 195 kV, 280 μA, and an FOV of 37.41 mm using Micro Focus TXS225UF (TESCO Corporation, Japan). The sheet consisted of an Al current collector, cathode, separator, graphite anode, and Cu current collector. The neutron transmission near the centre of the sample was approximately 27% and nearly uniform. The electrolyte level was measured on the left and right sides of the battery. Here, the cross-section of the battery sample taken via X-CT (Fig. 1a) clearly shows the jerry roll structure of the battery. The spatial resolution for neutron imaging, characterised using the line profile of the edge of the sample, was 248 µm (Fig. 4). Although this spatial resolution was larger than the thickness of the stacked electrode, as shown in Fig. 1c, the internal structure of the battery was sufficiently distinguishable. Note that the detectable structure in this study is lateral direction not the thickness direction of the layered structure.

**Figure 5:**
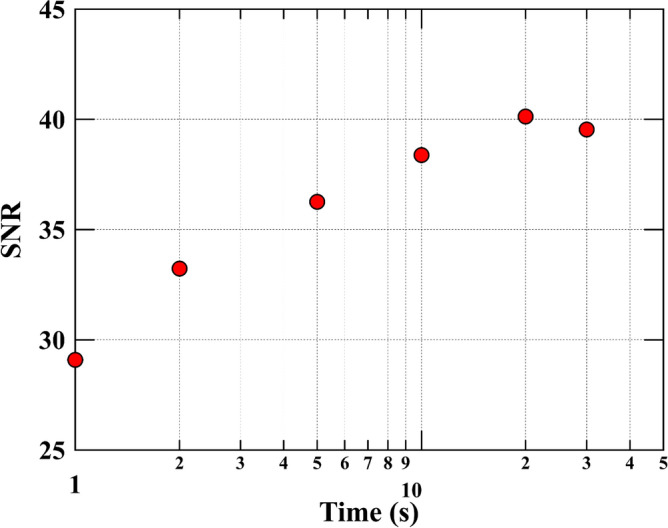
Evolution of the signal-to-noise ratio (SNR) with time. Variation of the SNR for the neutron transmission images as a function of the exposure time. The SNR increased with time.

Next, we evaluated the exposure time dependence of the SNR of the neutron transmission image (Fig. 5). In this study, the SNR was defined using the following equation:

2 $$SNR=10{\text{log}}_{10}\left(\frac{{I}_{\text{avg}}^{2}}{{\sigma }^{2}}\right),$$

where *I*_avg_ is the average intensity of the line profile in the neutron image and *σ* is the corresponding standard deviation. Considering the PP film and the separator are the thinnest materials in the battery, we established that an SNR of 30 can identify these components. An exposure time of 4.8 s, obtained by stacking 10 images with an exposure time of 0.48 s, achieved the target SNR. The accumulated image was binned to 1024 × 1024 pixels to improve statistics.

### Thermal abuse test

**Figure 6:**
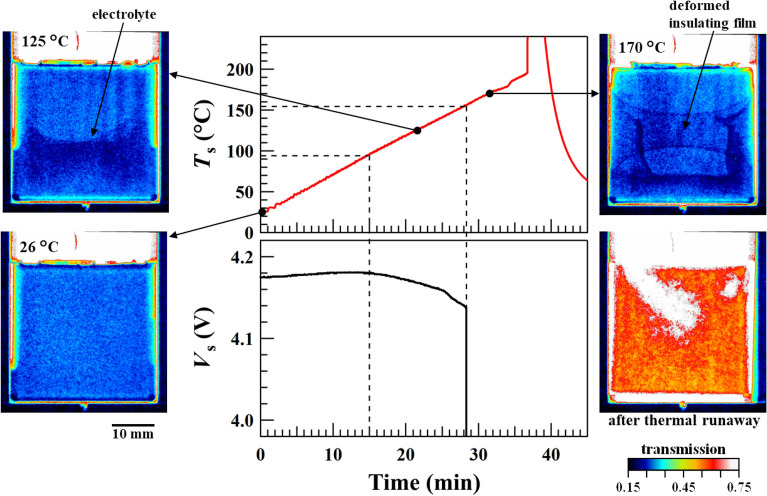
Temporal dependence of the voltage and temperature. Variations in the temperature and voltage of the battery sample as a function of time. Transmission images are shown at different temperatures with a visually distinguishable contrast.

Figure 6 shows the time dependence of the sample temperature (*T*_*s*_) and voltage (*V*_*s*_) as a function of heating time. The transmission images at different temperatures are also shown. The results indicate that the sample temperature increased almost linearly with time up to a temperature of approximately 190 °C and then increased steeply due to the thermal runaway, reaching 649 °C in 8 s (not shown in Fig. 6). The temperature decreased quickly following the steep increase owing to thermal runaway. The small step observed in *T*_s_ at approximately 185 °C was likely caused by the slight movement of the thermocouple due to thermal expansion. Simultaneously, the sample voltage at room temperature (26 °C) was measured at 4.175 V and remained stable within ± 0.005 V up to approximately 90 °C (t = 15 min). As *T*_s_ increased, the sample voltage *V*_s_ began to gradually decrease, showing a small kink at around *T*_s_ = 130 °C (t = 25 min) and then suddenly dropped down at *T*_s_ = 157 °C (t = 28 min). This sudden drop in *V*_s_ is caused by the disconnect of the electrical circuit in the battery, thus highlighting the so-called shutdown mechanism that prevents thermal runaway.

The continuously recorded neutron transmission images demonstrate the deformation of the internal structure, as shown in Fig. 6. The neutron transmission image changed significantly at 125 °C. A significant contrast was observed between the upper and lower parts of the battery, attributed to the electrolyte, which soaked into the separator, seeping out because of the shutdown of the separator and accumulating in the lower part of the battery.

### Heating to 170 °C

Figure 2b shows the heat-treated battery with the battery can removed. Upon heating the sample to 170 °C, the blue PP film shrank vertically by approximately one-third of its size (Fig. 2b). The deformed image exhibits almost the same texture as the neutron transmission image shown in Fig. 2c. Based on the time evolution of the neutron transmission images, the PP film deformed over approximately 120 s. As *T*_s_ and neutron transmission images did not change drastically even after deformation, we can conclude that the deformation of the PP film did not cause thermal runaway. After deformation, the shape of the PP film was preserved for approximately 7 min until just before thermal runaway. Furthermore, battery deformation was observed; the battery appeared to expand in thickness on both sides slightly, attributed to an increase in the internal pressure caused by the decomposition of the electrolyte. The difference of the transmission ∆*T* across the PP film was 2–3% (Fig. 2d). The thickness of the PP film was only 20 μm, almost the same as the separator thickness. As the PP film is not porous, its deformation behaviour was clearly observed, as shown in Fig. 6. However, the separator displayed no shrinkage over the entire heating test range, possibly because the separator was made of porous material; therefore, changes in transmittance could not be clearly observed, and the separator did not shrink until immediately before thermal runaway. After disassembling the system, we confirmed that heat treatment to 170 °C did not deform the separators.

**Figure 7:**
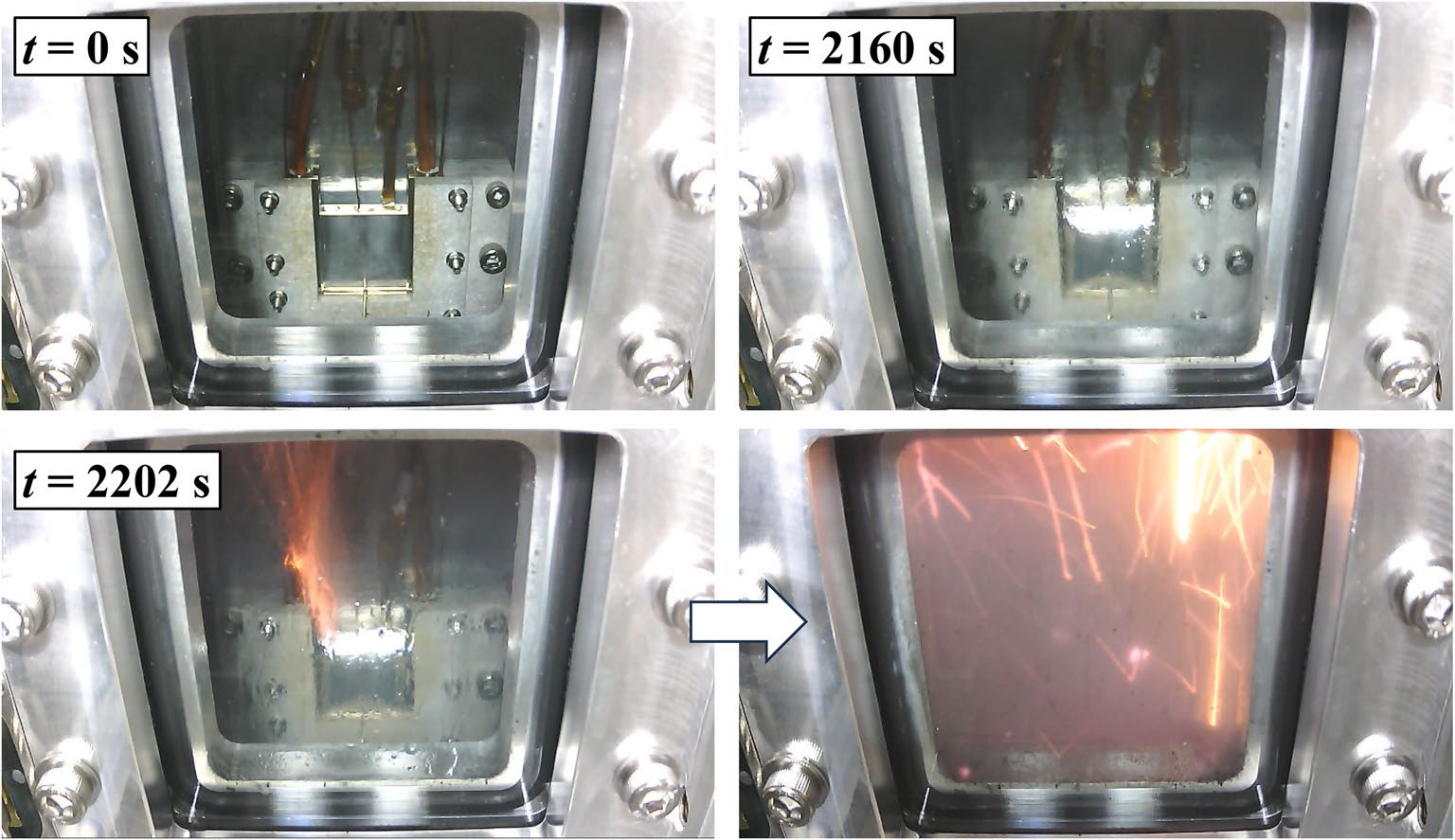
Snapshots of the battery sample inside the inner chamber. The battery sample is clearly seen in the inner chamber (*t* = 0 s). Before the thermal runaway (*t* = 2160 s), the liquid electrolyte is spewed from the safety valve and soaked the glass window. At *t* = 2202 s, sparks due to the thermal runaway erupted from the upper left of the battery sample and instantly spread throughout the inner chamber.

After thermal runaway, the transmission increased to approximately 0.6, indicating that the electrodes and electrolyte had spewed out of the battery (Fig. 7 and Video 1). Furthermore, an area from the centre of the battery to the upper left displayed higher transmission than in other areas, suggesting that the battery component spewed out from the safety valve as well, as shown in Fig. 1.

### Chamber fabrication

Another important aspect of this study is the setup, which ensured that no gas or particles leaked during the experiment to prevent contamination of the environment with radioactive materials. As discussed in the Supplementary Information, the liquid electrolyte spewed out from the top of the battery before thermal runaway because of the increase in the internal pressure. Additional high-temperature particles, such as carbonated materials, vapour electrolytes, and cobalt compounds, also spewed out intensely during thermal runaway. Using the airtight chamber developed in this study, the particles and gases were entirely confined inside the inner chamber (Video 1). After the thermal runaway, *T*_s_ dropped down to 40 °C in 20 min, while the internal pressure was at approximately 140 kPa for a month after the neutron experiment, thus demonstrating the airtightness performance of the proposed in situ neutron experiment.

## Conclusion

In this study, we developed an in situ heating system for use in neutron imaging in response to the significant demand for battery safety. We fabricated an airtight chamber to withstand the pressure and heat of the thermal runaway without blocking neutron transmission through the sample. Furthermore, an optimised heating method was adopted to obtain a clear neutron image and smoothly heat the sample. We succeeded in observing the internal structure of the battery sample until thermal runaway, with a lower limitation of visibility of ∆*T* ~ 2%. We developed an in situ neutron imaging system that enabled observing nearly all internal structural deformations of the battery with a time resolution of a few seconds. In the future, we plan to measure the internal structural changes in various degraded batteries using neutron radiography with this chamber up to thermal runaway and reveal the relationship between the internal structural changes and the mechanism of thermal runaway for battery safety.

### Supplementary Information

Supplementary Legends.

Supplementary Video 1.

## Data Availability

The data that support the findings of this study are available from the corresponding author upon reasonable request.

## References

[1] McGovern ME (2023). A review of research needs in nondestructive evaluation for quality verification in electric vehicle lithium-ion battery cell manufacturing. J. Power Sources.

[2] Guo X (2023). Intelligent monitoring for safety-enhanced lithium-ion/sodium-ion batteries. Adv. Energy Mater..

[3] Janek J, Zeier WG (2023). Challenges in speeding up solid-state battery development. Nat. Energy.

[4] Brunetaud R (2023). Non-destructive state-of-health diagnosis algorithm for blended electrode lithium-ion battery. J. Energy Storage.

[5] Wassiliadis N, Kriegler J, Gamra KA, Lienkamp M (2023). Model-based health-aware fast charging to mitigate the risk of lithium plating and prolong the cycle life of lithium-ion batteries in electric vehicles. J. Power Sources.

[6] Chen Y (2021). A review of lithium-ion battery safety concerns: The issues, strategies, and testing standards. J. Energy Chem..

[7] Yokoshima T (2018). Direct observation of internal state of thermal runaway in lithium ion battery during nail-penetration test. J. Power Sources.

[8] Deng Z (2021). Recent progress on advanced imaging techniques for lithium-ion batteries. Adv. Energy Mater..

[9] Mallick S, Gayen D (2023). Thermal behaviour and thermal runaway propagation in lithium-ion battery systems—A critical review. J. Energy Storage.

[10] Hatchard TD, MacNeil DD, Basu A, Dahn JR (2001). Thermal model of cylindrical and prismatic lithium-ion cells. J. Electrochem. Soc..

[11] Kim G-H, Pesaran A, Spotnitz R (2007). A three-dimensional thermal abuse model for lithium-ion cells. J. Power Sources.

[12] Kondo H, Baba N, Makimura Y, Itou Y, Kobayashi T (2020). Model validation and simulation study on the thermal abuse behavior of LiNi_0.__8_Co_0.__15_Al_0.__05_O_2_-based batteries. J. Power Sources.

[13] Finegan DP (2015). In-operando high-speed tomography of lithium-ion batteries during thermal runaway. Nat. Commun..

[14] Finegan DP (2018). Identifying the cause of rupture of Li-ion batteries during thermal runaway. Adv. Sci..

[15] Patel D, Robinson JB, Ball S, Brett DJL, Shearing PR (2020). Thermal runaway of a Li-ion battery studied by combined ARC and multi-length scale X-ray CT. J. Electrochem. Soc..

[16] Finegan DP (2016). Investigating lithium-ion battery materials during overcharge-induced thermal runaway: An operando and multi-scale X-ray CT study. Phys. Chem. Chem. Phys..

[17] Finegan DP (2017). Characterising thermal runaway within lithium-ion cells by inducing and monitoring internal short circuits. Energy Environ. Sci..

[18] Pham MTM (2020). Correlative acoustic time-of-flight spectroscopy and X-ray imaging to investigate gas-induced delamination in lithium-ion pouch cells during thermal runaway. J. Power Sources.

[19] Lanz M, Lehmann E, Imhof R, Exnar I, Novák P (2001). In situ neutron radiography of lithium-ion batteries during charge/discharge cycling. J. Power Sources.

[20] Goers D (2004). In situ neutron radiography of lithium-ion batteries: The gas evolution on graphite electrodes during the charging. J. Power Sources.

[21] Michalak B (2015). Gas evolution in operating lithium-ion batteries studied in situ by neutron imaging. Sci. Rep..

[22] Siegel JB, Stefanopoulou AG, Hagans P, Ding Y, Gorsich D (2013). Expansion of lithium ion pouch cell batteries: Observations from neutron imaging. J. Electrochem. Soc..

[23] Ma T (2020). Degradation mechanism study and safety hazard analysis of overdischarge on commercialized lithium-ion batteries. ACS Appl. Mater. Interfaces.

[24] Shinohara T (2020). The energy-resolved neutron imaging system. RADEN. Rev. Sci. Instrum..

[25] Matsumoto Y (2017). Recent progress of radiography and tomography at the energy-resolved neutron imaging system RADEN. Phys. Procedia.

[26] Schneider CA, Rasband WS, Eliceiri KW (2012). NIH Image to ImageJ: 25 years of image analysis. Nat. Methods.

