# Supplementary material for: In situ neutron imaging of lithium-ion batteries during heating to thermal runaway
Source: Sci Rep. 2023 Dec 12;13:22082. doi: 10.1038/s41598-023-49399-1 (PMC10716412; doi:10.1038/s41598-023-49399-1)
Supplement: Supplementary file 1 — Supplementary Legends. [file 41598_2023_49399_MOESM1_ESM.docx]

Supplementary Information

**Movie S1**: The internal view of the inner box using the webcam. This video started before 10 seconds from the thermal runaway.
